# Crude Extracts and Alkaloids Derived from *Ipomoea-Periglandula* Symbiotic Association Cause Mortality of Asian Citrus Psyllid *Diaphorina citri* Kuwayama (Hemiptera: Psyllidae)

**DOI:** 10.3390/insects12100929

**Published:** 2021-10-12

**Authors:** Xue-Dong Chen, Navneet Kaur, David R. Horton, W. Rodney Cooper, Jawwad A. Qureshi, Lukasz L. Stelinski

**Affiliations:** 1 Citrus Research and Education Center, Entomology and Nematology Department, University of Florida, 700 Experiment Station Rd, Lake Alfred, FL 33850, USA; stelinski@ufl.edu; 2 Southwest Florida Research and Education Center, Entomology and Nematology Department, University of Florida, 2685 SR 29 North, Immokalee, FL 34142, USA; jawwadq@ufl.edu; 3 Department of Crop and Soil Science, Oregon State University, 3050 Campus Way, 107 Crop Science Building, Corvallis, OR 97331, USA; Navneet.Kaur@oregonstate.edu; 4 USDA-ARS, Temperate Tree Fruit and Vegetable Research Unit, Wapato, WA 98951, USA; david.horton@usda.gov (D.R.H.); rodney.cooper@usda.gov (W.R.C.)

**Keywords:** Convolvulaceae, ergot alkaloids, *Diaphorina citri*, mortality, citrus greening

## Abstract

**Simple Summary:**

Fungi in the genus *Periglandula* (Clavicipitaceae) are endosymbionts of plants in the Convolvulaceae family (morning glories and relatives) where they may help protect plants from herbivory by production of bioactive compounds known as ergot alkaloids. We investigated mortality and behavior of nymphs and adults of Asian citrus psyllid *Diaphorina citri* Kuwayama (Hemiptera: Psyllidae) exposed to crude extracts from morning glories and to synthetic ergot alkaloids known to be produced in Convolvulaceae-*Periglandula* symbioses. We monitored effects of extracts or synthetic compounds on survival, host settling, and feeding. Several ergot alkaloids reduced survival of *D. citri* on treated surfaces. Crude extracts and synthetic ergot alkaloids reduced *D. citri* adult settling on treated host plants compared with water controls. We observed an antifeedant effect of the crude extracts at concentrations which otherwise caused minimal adult mortality. Our results indicate that ergot alkaloids produce both toxic and sub-lethal effects on *D. citri* that could be useful for management of this pest.

**Abstract:**

Asian citrus psyllid *Diaphorina citri* Kuwayama (Hemiptera: Psyllidae) is an important economic pest of citrus crops because it vectors the causal pathogen of huanglongbing (HLB; aka citrus greening). Population suppression of *D. citri* with insecticides has been disproportionally relied on for HLB management and a greater diversity of more sustainable tools is needed. *Periglandula* spp. is a fungal endosymbiont (family Clavicipitaceae) that forms a mutualistic relationship with members of plants in family Convolvulaceae. This association results in the production of ergot alkaloids that were previously documented as having psyllicidal properties. We investigated the mortality and behavior of *D. citri* exposed to crude extracts from morning glories in the plant family Convolvulaceae, as well as synthetic ergot alkaloids. Nymphs and adults were exposed to the crude plant extracts from *Periglandula* positive species of Convolvulaceae, as well as five synthetic ergot alkaloids. Treatments were prepared by exposing clippings of citrus to 100 ng/µL of crude extract from *Periglandula*-positive species of *Ipomoea* (*I. imperati*, *I. leptophylla*, *I. pandurata* and *I. tricolor*), and *Turbina corymbosa*, and from one *Periglandula*-negative species (*I. alba*) (100 ng/µL). Mortality of adult and nymphal *D. citri* was significantly higher than the control after exposure to extracts from *I. tricolor* and *I. imperati*. The synthetic ergot alkaloids, lysergol (10–100 ng/µL), ergonovine maleate (100 ng/µL), agroclavine (10–100 ng/µL), and ergosine (10–100 ng/µL) increased mortality of *D. citri* nymphs, while ergosine (100 ng/µL) and agroclavine (100 ng/µL) increased mortality of adults compared to water controls. Fewer *D. citri* adults settled on plants treated with crude extracts or synthetic ergot alkaloids than on water controls at 48 h after release. *D. citri* that fed on citrus leaves treated with 10 ng/μL solution of crude extract from the *Periglandula*-positive species *Ipomoea* (*I. imperati*, *I. leptophylla*, *I. pandurata*, *I. tricolor)*, and *Turbina corymbosa* excreted significantly less honeydew compared with a negative water control and extract from *Periglandula*-negative species (*I. alba*). Our results indicate that crude extracts and ergot alkaloids exhibit toxic and sub-lethal effects on *D. citri* that could be useful for management of this pest.

## 1. Introduction

Huanglongbing (HLB) or citrus greening is a disease that limits production of commercially important *Citrus* spp. worldwide [1]. Three taxonomic species of pathogenic bacteria are associated with HLB: *Candidatus* Liberibacter asiaticus, *C.* Liberibacter africanus and *C.* Liberibacter americanus. In Florida, only *C.* Liberibacter asiaticus (*C*Las) has been reported and is transmitted by the vector, Asian citrus psyllid, *Diaphorina citri* Kuwayama [1,2]. Despite efforts to control the disease and/or vector, current strategies have had limited effect on curtailing disease spread resulting in lost commercial feasibility. Florida’s citrus industry has been devastated, losing 74% of production since 2005 [3], putting the future of America’s citrus production capabilities at risk. Insecticidal suppression of *D. citri* [4,5,6] has to date played a disproportionally large role in HLB management. Conventional spray application methods of high volume airblast sprays of >100 GPA are often insufficient to manage the need for 8–12 applications that target *D. citri* alone [7,8]. During leaf flushing, additional sprays of selective insecticides are further applied in an effort to reduce nymphs [7]. Both soil and foliar insecticides are applied and at times rotation has not been practiced [9]. Biological control may also play a role in population reduction of *D. citri*, but it has been incompatible with intensive insecticide use [10,11,12]. Therefore, further effort is warranted to identify a greater diversity of more sustainable tools to improve HLB management.

Recent studies with potato psyllid, *Bactericera cockerelli* (Šulc) showed that this pest species exhibits egg-to-adult development on several species of morning glories in the family Convolvulaceae but dies rapidly on other species in the same genus [13]. The members of plant family Convolvulaceae have long been known to associate with Claviciptaceous fungi *Periglandula* spp. and to harbor ergot alkaloids produced by the fungal associate [14,15]. Ergot alkaloids are specialized metabolites derived from tryptophan and are produced by several fungi representing different phylogenetic lineages and different ecological niches [16,17]. Ergot alkaloids are generally categorized into one of the three groups (clavines, lysergic acid amides, and ergopeptines) based upon structural characteristics of the tetracyclic ergoline ring common to this class of compounds [18,19]. Ergot alkaloid profiles differ extensively among morning glory taxa [13,20,21,22,23]. Previous studies conducted by Kaur et al. demonstrated that newly hatched potato psyllid nymphs rapidly died on plants harboring *Periglandula* and ergot alkaloids, but often survived on species lacking the fungus and associated ergot alkaloids [13,20,24]. These results provided correlative evidence that *Periglandula* protects Convolvulaceae from insect herbivores by producing alkaloids having insecticidal properties.

The purpose of this study was to investigate the toxicity and sublethal effects of crude extracts from Convolvulaceae (morning glory) species as well as synthetic ergot alkaloids against adult and nymphal *D. citri*.

## 2. Materials and Methods

### 2.1. Insects

A laboratory population of *D. citri* was reared in a greenhouse at the Citrus Research and Education Center (CREC), University of Florida, Lake Alfred, FL. The culture was established in 2000 from field-collected insects in Polk County, Florida (27.86° N, 81.69° W) prior to the discovery of HLB in the state and this strain has been reared without exposure to insecticide or subsequent input of field-collected *D. citri*. *C*Las-negative *D. citri* were confirmed by testing a random sub-sample of *D. citri* and plants monthly using a quantitative real-time polymerase chain reaction (qPCR) assay [25]. The colony was maintained on sweet orange [*Citrus sinensis* (L.) Osbec] ‘Valencia’ in a temperature-controlled greenhouse at 27–28 °C, with 60–65% relative humidity and a 14:10 h (light:dark) photoperiod.

### 2.2. Plant Materials

Plants (height: 60 cm) were purchased from a local nursery (Dundee, FL, USA) certified free of *C*las and housed in a greenhouse at the CREC in Lake Alfred FL, at 27–28 °C, 60–65% RH, and a 14:10 h (light: dark) photoperiod. Uninfected plants used in experiments were 1–2-year-old Swingle citrumelo (*Citrus paradise* × *Poncirus trifoliata*) and *Citrus reshni* hort. ex Tanaka (potted trees; height: 15–20 cm). The nursery-obtained plants were confirmed negative for *C*Las infection by qPCR as described in Pelz-Stelinski et al. [25] and Chen et al. [26].

### 2.3. Crude Extract Bioassay

Crude extracts were obtained from Convolvulaceae plants grown and maintained in greenhouse at the USDA-ARS, Temperate Tree Fruit and Vegetable Research Unit, Wapato, WA, USA according to the protocol described in Kaur et al. [24]. The plants (Convolvulaceae) used in this investigation included both *Periglandula*-positive species of *Ipomoea* (*I. imperati*, *I. leptophylla*, *I. pandurata*, *I. tricolor*) and *Turbina corymbosa*, as well as one *Periglandula*-negative species (*I. alba*), which served as an internal negative control. Crude extracts were prepared from all plants according to the protocol described in Kaur et al. [24]. Air-dried leaf materials were ground in 95% HPLC grade methanol (Thermo Fisher Scientific, Waltham, MA, USA) and homogenate was subjected to filtration through a 0.2 µm HT Tuffyrn Membrane (Pall Corporation, Port Washington, NY, USA). Upon methanol evaporation, the filtrate was reconstituted using 1 mL of water. Quantification of ergot alkaloids was performed using LC-MS/MS procedures described in Kaur et al. [24]. The crude extracts were stored in amber vials at 4 °C until insect bioassays were performed.

Young leaves (soft, immature, and fully expanded) (Length: 7.16 ± 0.04 cm; Wide: 3.68 ± 0.09 cm; Stem > 1.5 cm) from citrus were treated by immersing the cut ends of clipping in 1 mL extract solutions with 2 mL vials. Control vials contained water. Clipping immersed in solution treatments were housed within plastic solo-cups (1 1/4 oz 37.0 mL) (Solo Cup Company, Highland Park, IL, USA) covered with vented lids. A small hole was made at the bottom of each cup to accommodate the stem end of clipping. A hole of similar size was made in the lid of each 2 mL plastic vial and vials were glued to bottoms of the plastic cups. The cut end of each clipping was fully immersed in solution within each vial. Parafilm was wrapped around the stem of each clipping at the cup-vial junction to minimize physical damage to the stem and to prevent insect escape. Assays were conducted in environmentally controlled rooms with temperature set to 25 °C and a light: dark cycle of 16 h:8 h. Nymphs (4–5th instar stage) and adults of *D. citri* were collected from cultures using a small paint brush and placed onto single leaflets of each treated plant. Five *D. citri* adults or five nymphs were exposed to treatments per replicate and all treatments were replicated four times. The number of living and dead psyllids was recorded every 24 h for 3 d for nymphs and 4 d for adults. Psyllids were considered dead if appendages did not respond upon prodding with a camel-hair brush.

### 2.4. Synthetic Alkaloid Bioassay

Five alkaloids known to be produced by *Periglandula-Convolvulaceae* association were examined in assays. The alkaloids included one clavine (agroclavine), one lysergol acid amide (lysergol), and three ergopeptines (ergonovine maleate, ergocornine and ergosine). Agroclavine, ergocornine, and ergosine were obtained from Romer Labs (Newark, DE, USA). Lysergol was obtained from Toronto Research Chemicals (North York, ON, Canada), while ergonovine maleate was procured from Sigma-Aldrich (St. Louis, MO, USA). Treatments were examined in dose-response assays consisting of four concentrations of each compound (0, 1, 10, and 100 ng/µL) in 2 mL of distilled water. The toxicity bioassay methods were conducted as described above (Supplementary Data Figure S1).

### 2.5. Effect of Crude Extracts and Synthetic Alkaloids on Settling Behavior of D. citri

To investigate the settling behavior of *D. citri* on alkaloid treated versus non-treated Swingle citrumelo (*Citrus paradisi* × *Poncirus trifoliata*) and *C. reshni* seedlings, adult psyllids were given a choice between untreated seedlings and similar plants treated with one concentration of each crude extract or synthetic alkaloid prepared as described above; the concentrations were known to be sub-lethal based on results of the toxicity assays (see Results). Extracts examined for potential repellency in this experiment were limited to *Periglandula*-positive Convolvulaceae species. Treated seedlings were sprayed with 10 mL of crude extract solution or synthetic alkaloid at a concentration of 10 ng/µL dissolved in water, while control seedlings were sprayed with 5 mL of water only using a handheld atomizer (The Bottle Crew, West Bloomfield, MI, USA). Subsequently, treated and control plants (potted trees; height: 15–20 cm) were introduced into 30 cm × 30 cm × 30 cm insect cages (#1466CV, BioQuip Products, Rancho Dominguez, CA, USA). The two potted plants (alkaloid-treated versus control) were positioned in opposite corners of each cage and their position was chosen at random. Fifty mixed-sex *D. citri* were released from containers in the center of each cage. The number of *D. citri* settling per plant was recorded 48 h after release. There were two biological replicates (insect cages) for each crude extract or synthetic alkaloid treatment during each run of the experiment and two independent runs with each treatment were conducted on different days. The entire experiment was repeated twice.

### 2.6. Antifeedant Effect of Crude Extracts and Synthetic Alkaloids as Measured by Honeydew Excretion

The objective of this experiment was to determine whether crude extracts from *Ipomoea* species or synthetic alkaloids affect feeding of *D. citri* adults as quantified by honeydew excretion. *D. citri* adults were exposed to leaves treated with 0.5 mL of each crude extract or synthetic alkaloid at a 10 ng/µL concentration using a handheld atomizer. Leaf discs (35 mm diameter) were cut from citrus leaves collected from Swingle citrumelo trees that were not treated with insecticide. Five adult *D. citri* of mixed age and sex were released into each dish, which were sealed with lids lined with 35 mm Whatman filter paper (Whatman international Ltd., Kent, UK). Petri dishes were placed upside down to collect honeydew droplets onto the filter paper. Dishes were maintained at 25 ± 2 °C, 50 ± 5% RH and 14:10 h light: dark photoperiod for 48 h. Adult mortality in this experiment was very low (5% at the highest concentration); therefore, data from both healthy and dead/dying *D. citri* were included in the analysis. Collected filter paper discs were subjected to a ninhydrin (Sigma-Aldrich, SR Louis, MO, USA) test to facilitate counting of honeydew droplets. Honeydew droplets were counted 48 h after transfer into growth chambers. Each plant species crude extract and synthetic alkaloid treatment was replicated four times and 10 insects were tested per replicate. The entire experiment was repeated two times on different dates.

### 2.7. Statistical Analysis

Mortality of adult and nymph *D. citri* was compared among treatments by logistic regression using the GLIMMIX procedure of SAS 9.4. The response variable (numbers dead/numbers assayed) was modeled using an ‘events/trials’ syntax while specifying a logit function. The MODEL statement included the DDFM=NONE and CHISQ options to specify chi square statistics. Separate analyses were run for each compound comparing mortality caused by commercial alkaloids with time (24, 48, 72, and 96 h), concentration (0, 1, 10, and 100 ng/µL), and the time by concentration interaction included as fixed effects. Assays testing mortality caused by crude alkaloid extracts were conducted at one concentration with water as a control. The fixed effects included time, treatment (crude extract), and the time by treatment interaction. Each analysis was conducted as repeated measures by including REP(TIME) as a RANDOM variable. When overall chi square analyses indicated significant differences among treatments, the SLICEDIFF option of the LSMEANS statement was used for comparisons among simple effects. The CL and ILINK options were included in the LSMEANS statement to include Wald 95% confidence intervals and back-transformed proportions, respectively.

Adult settling behavior was analyzed by logistic regression using PROC GLIMMIX of SAS 9.4 with the events/trials syntax (number of adults on a plant/number of adults released into the cage). Run was included as a RANDOM variable, and the SUBJECT=CAGE was included in the RANDOM statement to designate cage as the biological replicate.

The number of honeydew droplets produced by adult *D. citri* feeding on leaves treated with crude extracts or commercial alkaloids was compared among treatments using the GLIMMIX procedure. In both analyses, treatment was included as a fixed effect and REP(TRIAL) was included as a RANDOM variable. The ADJUST=SIMULATE option of the LSMEANS statement was used to compare simple effects when the overall ANOVA indicated significant differences among means.

## 3. Results

### 3.1. Crude Extract Bioassay

There was a time by treatment interaction for nymphal mortality among crude extract leaf treatments (Table 1: *df* = 10; *Χ*^2^ = 488; *p* < 0.001). In general, the highest mortality rates were observed among nymphs exposed to leaves treated with crude extracts from ergot-harboring species (*I. leptophylla*, *I. imperati*, *I. pandurata*, *T. corymbosa*, and *I. tricolor*), while the lowest mortality rates were observed among nymphs exposed to untreated leaves or leaves treated with extracts from the *Periglandula*-negative *I. alba* (Table 1).

There were also significant differences in adult mortality rates among crude extract treatments (Table 2: *df* = 6; *Χ*^2^ = 399.9; *p* < 0.001). Mortality rates did not differ with increasing exposure (*Χ*^2^ = 1.1; *df* = 3; *p =* 0.771), and the lack of a time by treatment interaction (*df* = 16; *Χ*^2^ = 8.9; *p* = 916) indicated that the effects of crude extract treatment on adult mortality were consistent over time. While the effects of crude extracts on adult mortality were not as great as those observed on nymph mortality, extracts from ergot-harboring species generally caused higher mortality than did the water control or extracts from *I. alba* (Table 2).

### 3.2. Synthetic Alkaloid Bioassay

**Figure 1:**
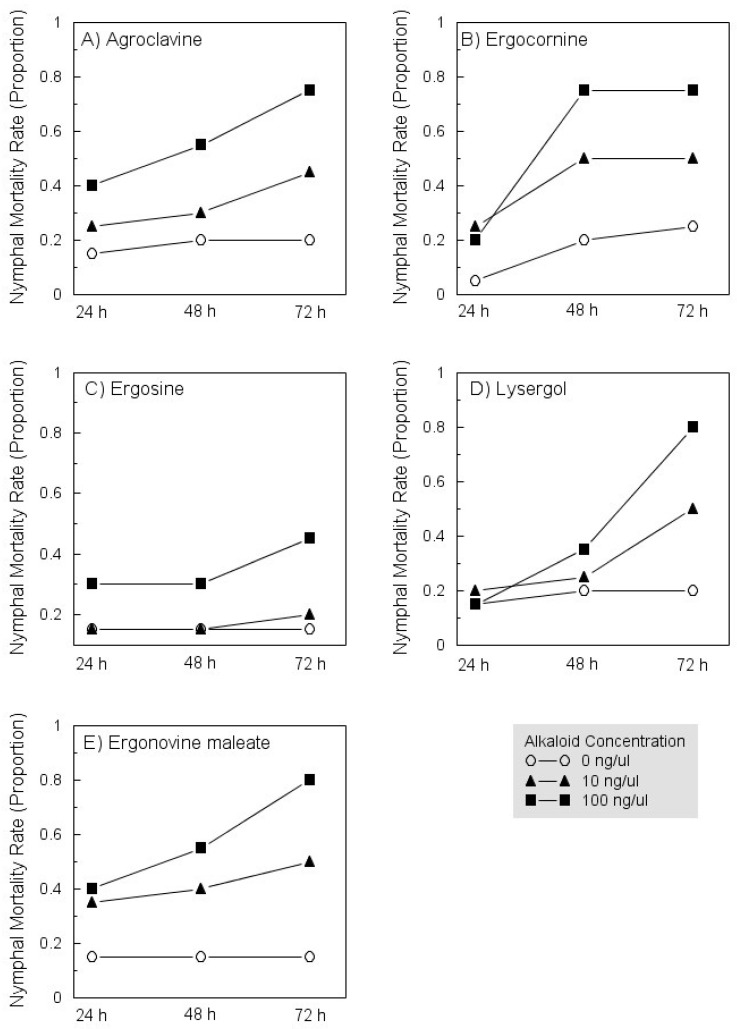
Exposure-dependent mortality rates among *Diaphornia citri* nymphs exposed to leaves treated with increasing concentrations of agroclavine (**A**), ergocornine (**B**), ergosine (**C**), lysergol (**D**), or ergonovine maleate (**E**). See Table 3 for statistical results. The figure depicts morality observed with the control (0 ng/µL) and at the two highest concentrations tested, 10 and 100 ng/µL.

There were significant differences in mortality rates among *D. citri* nymphs exposed to leaves treated with 0, 1, 10, or 100 ng/µL concentrations of most synthetic alkaloids except lysergol (Table 3). The lack of significant time by concentration interactions indicates that the effects of alkaloid treatments on nymph mortality rates were statistically constant across increasing exposure times (Table 3). In general, all compounds contributed to mortality of nymphs in a dose-dependent manner, and ergot-induced mortality tended to increase with time after initial exposure to treated leaves (Figure 1).

**Figure 2:**
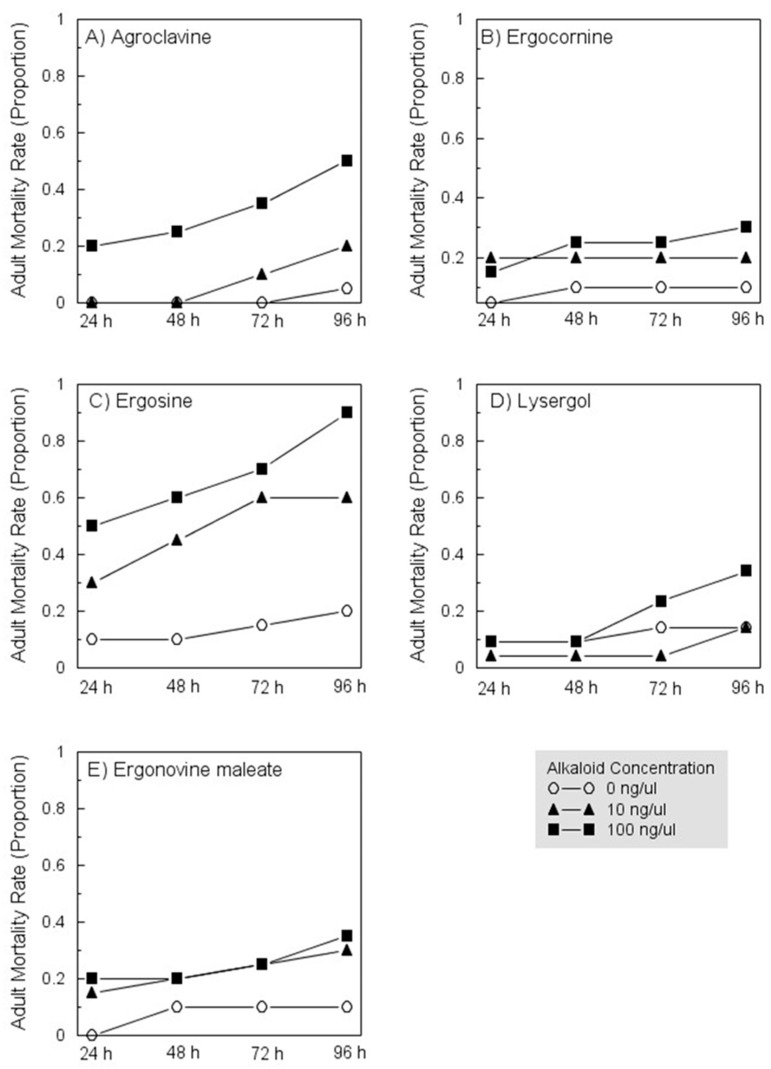
Exposure-dependent mortality rates among *Diaphornia citri* adults exposed to leaves treated with increasing concentrations of agroclavine (**A**), ergocornine (**B**), ergosine (**C**), lysergol (**D**), or ergonovine maleate (**E**). See Table 3 for statistical results. The figure depicts morality observd with the control (0 ng/µL) and at the two highest concentrations tested, 10 and 100 ng/µL.

Exposure to ergocornine- and ergosine-treated leaves led to a significant increase in adult mortality. Mortality rates did not differ among adults exposed to leaves treated with the other compounds, although there was a suggestion (*p* = 0.067) that mortality increased with concentration of agroclavine (Table 3; Figure 2). Numerical trends were generally consistent with those observed for nymphs, with dose-dependent mortality caused by exposure to each alkaloid, and with mortality rates increasing as time of exposure to compounds increased (Figure 2).

### 3.3. Effect of Crude Extracts and Synthetic Alkaloids on Host Settling and Feeding by D. citri

**Figure 3:**
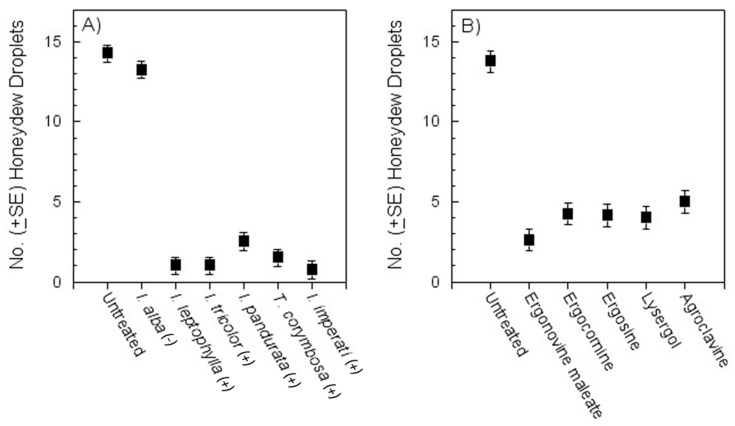
Effect of crude extracts from Convolvulaceae plant species (**A**) or synthetic alkaloids (**B**) on feeding by adult *Diaphorina citri* as measured by honeydew excretion.

Fewer *D. citri* adults settled on plants treated with crude plant extracts or with synthetic ergot alkaloids than on water controls at 48 h after release, regardless of treatment (Table 4). As measured by honeydew excretion, feeding by *D. citri* was significantly reduced on crude extract-treated leaves as compared with the *Periglandula*-negative (*I. alba*) and water controls (*df =* 6, 42; *F* = 120.4; *p* < 0.001) (Figure 3A). Feeding by *D. citri* was also significantly reduced on synthetic ergot alkaloid-treated leaves as compared negative water control leaves (*df* = 5, 35; *F* = 35.6; *p* < 0.001 (Figure 3B).

## 4. Discussion

This study presents experimental evidence that ergot alkaloids induced mortality of *D. citri.* These results are congruent with the hypothesis that alkaloids originating from the *Periglandula*-Convolvulaceae symbioses evolved from selection pressure imposed by herbivory. There is a need to understand the mode(s) of action and specific chemistry of these compounds in order to develop ergot alkaloids into potential useful tools for psyllid management. With the exception of ergocornine, the ergot alkaloids examined here affected *D. citri* nymph feeding on citrus disks. In contrast, the alkaloids had little effect of adult survival; only two of 20 comparisons increased mortality compared with the control. At the concentrations of alkaloids tested, they appeared to function as antibiotics (ergonovine), with respect to their effects on *D. citri*.

Negative effects of claviciptitacepous endophytes on insects were first reported by Prestidge et al. [27] who observed that perennial ryegrass and other endophyte-infected grasses exhibit enhanced tolerance to many insects [27]. Plant tolerance or resistance to insects may arise from deterrent properties causing herbivores to avoid hosts or due to antibiosis, where insects feed on such hosts, but suffer from growth or development abnormalities, which ultimately reduces population growth. Prestidge et al. [27] first demonstrated a negative correlation between injury from Argentine stem weevil, *Listronotus bonariensis*, and the frequency of endophyte-infected perennial ryegrass plots with high levels of endophtyte infection; grasses with endophytes suffered less injury from sod webworms (*Crambus* spp.) and had fewer adults and eggs present than those with lower levels of infection. Similarly, Ahmad et al. [28] found increased tolerance of infected perennial ryegrass to the bluegrass billbug, *Sphenophorus paruulus*, in field trial with different cultivars. Kaur et al. found that presence of a fungal symbiont in Convolvulaceae (*Periglandula* spp.) reduced psyllid survival [13]. Horton et al. showed that even a Convolvulaceae-specialized psyllid fails to develop on the Periglandula positive species assayed, even while developing extremely well on *I. alba* [29]. No choice laboratory feeding experiments have consistently demonstrated that many grasses, infected by at least 10 different clavicipitaceous endophytes, are more toxic to and reduce survival, growth, and developmental rate of insects; examples include: crickets (*Acheta domesticus*) [30]; black cutworms (*Agrotis segetum*) [31]; chinch bugs (*Blissus Ieucopterus hirtus*) [32]; sod webworms (*Crambus* spp.) [33]; black beetles (*Heteronychus arator*) [30]; Argentine stem weevils (*Listronotus bonariensis*) [34]; milkweed bugs (*Onocopeltus fasciatus*) [35]; aphids (*Rhopalosiphum padi*) [36] and (*Schizaphis graminum*) [37]; bluegrass billbugs (*Sphenophorus parvuius*) [28]; fall armyworms (*Spodoptera frugiperda*) [30]; southern armyworms (*Spodoptera eridania*) [27]; flour beetles (*Tribolium castaneum*) [38]; corn flea beetles (*Chaetocnema pulicaria*) [38]; potato psyllid, *Bactericera cockerelli* (Šulc) [35]; and sharpshooters (*Draecuiacephaia antica*) [38].

The phloem-limited bacterium *Candidatus* Liberibacter asiaticus that causes HLB in citrus is rapidly and effectively transmitted by *D. citri* [25,39]. We found an antifeedant effect of the crude extracts from *Ipomoea* species at the 10 ng/µL concentration, which otherwise caused minimal adult mortality. Our results indicate that further studies are warranted utilizing techniques such as electrical penetration graph (EPG) monitoring to characterize the feeding behavior of *D. citri* on ergot-treated foliage more precisely. Other sublethal effects of synthetic alkaloids were observed by examining settling behavior of *D. citri.* All of the synthetic alkaloids reduced host settling response by *D. citri.* Reduced settling of *D. citri* adults on ergot alkaloid-treated trees should not only reduce direct damage, but also reduce pathogen acquisition and perhaps inoculation. Host avoidance by *D. citri* caused by synthetic alkaloid treatment may contribute to HLB management and this hypothesis warrants further testing [6,40].

## 5. Conclusions

Our investigation indicates that plants in the morning glory family (Convolvulaceae) have insecticidal activity against *D. citri*. Further studies are underway to determine the mode of action of both alkaloids that appeared to cause direct toxicity to *D. citri*. Alkaloid extracts and some of the dominant alkaloids from Convolvulaceae may have potential as future eco-friendly approaches for management of *D. citri*. In the context of pest management in agricultural production, botanical insecticides are suitable candidates for use in organic food production in industrialized countries but can also play a greater role in the production and postharvest protection of food in developing countries.

## Figures and Tables

**Table 1:** Time-dependent mortality rates (proportion) of *Diaphornia citri* nymphs exposed to leaves treated with crude extracts from Convolvulaceae plant species. (+) *Periglandula*-positive species; (−) *Periglandula* negative species.

Treatment	24 h ^1^	48 h	72 h
*Water*	0 ^b^	0.05 ± 0.049 ^b^	0.08 ± 0.049 ^b^
*Ipomoea alba* (−)	0 ^b^	0.05 ± 0.007 ^b^	0.09 ± 0.077 ^b^
*Ipomoea leptophylla* (+)	0.30 ± 0.104 ^a^	0.45 ± 0.112 ^a^	0.70 ± 0.107 ^a^
*Ipomoea tricolor* (+)	0.25 ± 0.990 ^a^	0.30 ± 0.105 ^ab^	0.5 ± 0.114 ^ab^
*Ipomoea pandurata* (+)	0.15 ± 0.082 ^a^	0.30 ± 0.105 ^ab^	0.45 ± 0.113 ^ab^
*Turbina corymbosa* (+)	0.10 ± 0.069 ^a^	0.25 ± 0.099 ^ab^	0.85 ± 0.084 ^a^
*Ipomoea imperati* (+)	0.35 ± 0.108 ^a^	0.50 ± 0.112 ^a^	0.80 ± 0.094 ^a^

^1^ Means within columns with different letters differ significantly according to Wald’s 95% confidence interval.

**Table 2:** Time-dependent mortality rates (proportion) of *Diaphornia citri* adults exposed to leaves treated with crude extracts from Convolvulaceae plant species. (+) *Periglandula*-positive species; (−) *Periglandula*-negative species.

Treatment	24 h	48 h	72 h	96 h	Overall ^1^
*Water*	0	0	0.1 ± 0.067	0.1 ± 0.067	<0.01 ^b^
*Ipomoea alba* (−)	0	0	0.2 ± 0.089	0.2 ± 0.089	<0.01 ^b^
*Ipomoea leptophylla* (+)	0.1 ± 0.067	0.15 ± 0.080	0.25 ± 0.097	0.30 ± 0.103	0.19 ± 0.048 ^a^
*Ipomoea tricolor* (+)	0.15 ± 0.080	0.25 ± 0.097	0.35 ± 0.107	0.60 ± 0.110	0.32 ± 0.057 ^a^
*Ipomoea pandurata* (+)	0.10 ± 0.067	0.20 ± 0.089	0.25 ± 0.097	0.45 ± 0.111	0.23 ± 0.051 ^a^
*Turbina corymbosa* (+)	0.25 ± 0.097	0.25 ± 0.097	0.25 ± 0.097	0.25 ± 0.097	0.25 ± 0.048 ^a^
*Ipomoea imperati* (+)	0.10 ± 0.067	0.15 ± 0.080	0.25 ± 0.097	0.30 ± 0.103	0.25 ± 0.048 ^a^

^1^ Means within columns with different letters differ significantly according to Wald’s 95% confidence interval.

**Table 3:** Effects of alkaloid concentration and time of exposure on mortality of Asian citrus psyllid, *Diaphorina citri*, nymphs.

Treatment	Nymphs	Adults
Ergonovine maleate	Time: *df* = 2; *Χ*^2^ = 3.3; *p* = 0.192 Concentration: *df* = 3; *Χ*^2^ = 35.5; *p <* 0.001 Interaction: *df* = 6; *Χ*^2^ = 2.9; *p* = 0.815	Time: *df* = 3; *Χ*^2^ = 0.6; *p* = 0.906 Concentration: *df* = 3; *Χ*^2^ = 2.3; *p =* 0.504 Interaction: *df* = 9; *Χ*^2^ = 1.1; *p* = 0.999
Ergocornine	Time: *df* = 2; *Χ*^2^ = 17.9; *p* < 0.001 Concentration: *df* = 3; *Χ*^2^ = 19.6; *p <* 0.001 Interaction: *df* = 6; *Χ*^2^ = 2.7; *p* = 0.845	Time: *df* = 3; *Χ*^2^ = 1.5; *p* = 0.692 Concentration: *df* = 3; *Χ*^2^ = 3.7; *p =* 0.011 Interaction: *df* = 9; *Χ*^2^ = 1.1; *p* = 0.999
Ergosine	Time: *df* = 2; *Χ*^2^ = 0.59; *p* = 0.555 Concentration: *df* = 3; *Χ*^2^ = 8.2; *p =* 0.042 Interaction: *df* = 6; *Χ*^2^ = 0.6; *p* = 0.996	Time: *df* = 3; *Χ*^2^ = 13.4; *p* = 0.004 Concentration: *df* = 41.1; *Χ*^2^ = 8.2; *p <* 0.001 Interaction: *df* = 9; *Χ*^2^ = 2.4; *p* = 0.983
Lysergol	Time: *df* = 2; *Χ*^2^ = 13.7; *p* = 0.001 Concentration: *df* = 3; *Χ*^2^ = 6.7; *p =* 0.081 Interaction: *df* = 6; *Χ*^2^ = 7.4; *p* = 0.288	Time: *df* = 3; *Χ*^2^ = 4.4; *p* = 0.229 Concentration: *df* = 3; *Χ*^2^ = 4.1; *p =* 0.253 Interaction: *df* = 9; *Χ*^2^ = 1.8; *p* = 0.995
Agroclavine	Time: *df* = 2; *Χ*^2^ = 1.2; *p* = 0.552 Concentration: *df* = 3; *Χ*^2^ = 17.9; *p <* 0.001 Interaction: *df* = 6; *Χ*^2^ = 7.8; *p* = 0.934	Time: *df* = 3; *Χ*^2^ = 0.1; *p* = 0.999 Concentration: *df* = 3; *Χ*^2^ = 7.2; *p =* 0.067 Interaction: *df* = 9; *Χ*^2^ = 0.4; *p* = 0.999

**Table 4:** Percentage of *Diaphorina citri* adults settling on control plants versus those treated with synthetic alkaloids 48 h after release.

Treatment	Settled on Control ^a^	Settled on Treated Plants	Logistic Regression
Crude Plant Extracts			
*I. leptophylla* (+)	0.61 ± 0.035	0.25 ± 0.031	*df* = 1; *Χ*^2^ = 48.9; *p* < 0.001
*I. tricolor* (+)	0.57 ± 0.035	0.30 ± 0.032	*df* = 1; *Χ*^2^ = 28.8; *p* < 0.001
*I. pandurata* (+)	0.52 ± 0.035	0.29 ± 0.032	*df* = 1; *Χ*^2^ = 20.6; *p* < 0.001
*T. corymbosa*(+)	0.59 ± 0.035	0.31 ± 0.033	*df* = 1; *Χ*^2^ = 30.8; *p* < 0.001
*I. imperati* (+)	0.64 ± 0.034	0.24 ± 0.030	*df* = 1; *Χ*^2^ = 59.4; *p* < 0.001
Synthetic Alkaloids			
Ergonovine maleate	0.74 ± 0.031	0.19 ± 0.027	*df* = 1; *Χ*^2^ = 106.5; *p* < 0.001
Ergosine	0.68 ± 0.033	0.25 ± 0.030	*df* = 1; *Χ*^2^ = 70.6; *p* < 0.001
Lysergol	0.72 ± 0.032	0.19 ± 0.028	*df* = 1; *Χ*^2^ = 98.5; *p* < 0.001
Agroclavine	0.73 ± 0.032	0.19 ± 0.028	*df* = 1; *Χ*^2^ = 101.68; *p* < 0.001

^a^: Sum of the proportion of psyllids that settled on control and treated plants may not equal 1 due to psyllids that settled on neither plant.

## Data Availability

The data presented in this study are available on request from the corresponding authors.

## Supplementary Materials

The following are available online at https://www.mdpi.com/article/10.3390/insects12100929/s1, Figure S1: Crude extracts and alkaloid of toxicity of bioassay procedure on Asian citrus psyllid, *Diaphorina citri*.
