# Supplementary material for: Crude Extracts and Alkaloids Derived from Ipomoea-Periglandula Symbiotic Association Cause Mortality of Asian Citrus Psyllid Diaphorina citri Kuwayama (Hemiptera: Psyllidae)
Source: Insects. 2021 Oct 12;12(10):929. doi: 10.3390/insects12100929 (PMC8539733; doi:10.3390/insects12100929)
Supplement: Supplementary file 1 [file insects-12-00929-s001.zip › insects-1394110-supplementary.pdf]

insects

Article

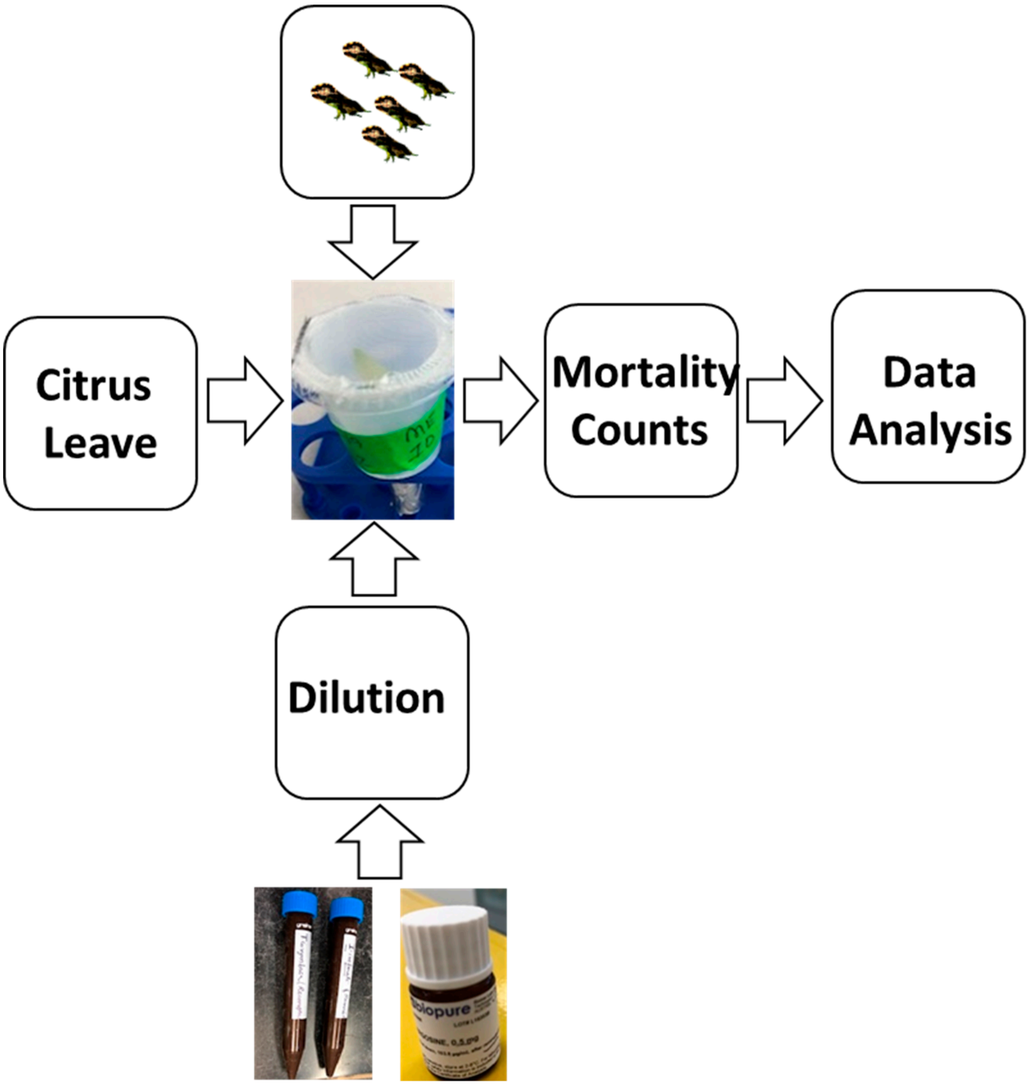

Supplementary Figure S1 . Crude extracts and alkaloid of toxicity of bioassay procedure on Asian citrus psyllid, *Diaphorina citri*.
